# Enhancing cellulose acetate biodegradability in cigarette filters: an in-depth analysis of thermal alkaline pretreatment, microbial dynamics, and breakdown pathway prediction

**DOI:** 10.1186/s12934-024-02476-0

**Published:** 2024-07-18

**Authors:** Darsha Prabhaharan, Hyojung Park, Okkyoung Choi, Amith Abraham, Byoung-In Sang

**Affiliations:** 1 Department of Chemical Engineering, Hanyang University, 222 Wangsimniro, Seongdong-gu, Seoul, 04763 Republic of Korea; 2 Center of Convergence Bioceramic Materials, Korea Institute of Ceramic Engineering & Technology, 202, Osongsaengmyeong 1-ro, Osong-eup, Heungdeok-gu, Cheongju-si, Chungcheongbuk-do Republic of Korea; 3 Eco Lab Center, SK Ecoplant, 51, Jong-ro, Jongno-gu, Seoul, Republic of Korea

**Keywords:** Cellulose acetate, Anaerobic digestion, Biodegradability, Thermal alkaline pretreatment

## Abstract

**Background:**

The demand for bioplastics has increased exponentially as they have emerged as alternatives to petrochemical plastics. However, there is a substantial lack of knowledge regarding bioplastic degradation. This study developed a novel pretreatment method to improve the accessibility of a bioplastic substrate for biodegradation. In this study, cellulose acetate, a bioplastic found in the world’s most littered waste, e.g. cigarette filters, was selected as a potential substrate. Before anaerobic digestion, three thermal alkaline pretreatments: TA 30 °C, TA 90 °C, and TA 121 °C, were used to evaluate their effects on the chemical alterations of cellulose acetate.

**Result:**

The ester groups in cellulose acetate were significantly reduced by the TA 30 °C pretreatment, as seen by a decrease in C = O stretching vibrations and shortening of C − O stretches (1,270 ∼ 1,210 cm^− 1^), indicating effective removal of acetyl groups. This pretreatment significantly enhanced cellulose acetate biodegradability to a maximum of 91%, surpassing the previously reported cellulose acetate degradation. Methane production increased to 695.0 ± 4 mL/g of volatile solid after TA 30 °C pretreatment, indicating enhanced cellulose acetate accessibility to microorganisms, which resulted in superior biogas production compared to the control (306.0 ± 10 mL/g of volatile solid). Diverse microbes in the anaerobic digestion system included hydrolytic (*AB240379_g*,* Acetomicrobium*,* FN436103_g*, etc.), fermentative, and volatile fatty acids degrading bacteria (*JF417922_g*,* AB274492_g*,* Coprothermobacter*, etc.), with *Methanobacterium* and *Methanothermobacter* being the sole hydrogenotrophic methanogens in the anaerobic digestion system. Additionally, an attempt to predict the pathway for the effective degradation of cellulose acetate from the microbial community in different pretreatment conditions.

**Conclusions:**

To the best of our knowledge, this is the first study to estimate the maximum cellulose acetate degradation rate, with a simple and cost-effective pretreatment procedure. This approach holds promise for mitigating the environmental impact of cellulose acetate of cigarette filters and presents a sustainable and economically viable waste management strategy.

## Background

Growing concerns over global plastic pollution have led to increased use of biodegradable bioplastics [1]. The market for biodegradable bioplastics is predicted to grow by 50% by 2025 [2]. The global bioplastic market was valued at USD 15.3 billion in 2024 and is expected to grow to USD 45.2 billion by 2029, indicating a compound annual growth rate (CAGR) of 24.2% during this period. Despite the growing demand, the biodegradability and overall waste management of bioplastics are still being questioned. The complete degradation of bioplastics through any treatment process under aerobic and anaerobic conditions is not always guaranteed due to the difference between industrial and laboratory practices [3]. Moreover, most treatment plants are not specifically equipped for bioplastic processing, potentially leading to the contamination of digestate with macro and microplastic molecules, posing environmental hazards. A noteworthy example is Cellulose acetate (CA), derived from cellulose. Cellulose, sourced from sugarcane, wood, cotton, and recycled papers, can be converted into a polysaccharide ester like CA, which has become popular due to its desirable properties and sustainability [4, 5]. Cellulose is modified via acetylation, in which acetyl groups (-COCH3) replace hydrogen in the hydroxyl (-OH) groups of cellulose molecules to form CA. With efficient mechanical and thermal resistance, CA is utilized in diverse manufacturing practices, including syringe filters, photographic films, and cigarette filters (CF) [6].

Our attempt to degrade CA from CF is of great value due to three key reasons: environmental benefits, advancement in waste management, and economic benefits in the AD system. CF were originally designed to prevent toxins, including nicotine, arsenic, and lead from entering the smoker’s body, all of which are lethal for all living organisms. Unfortunately, poor disposal practices led to a global accumulation of approximately 1.2 million tons CF annually, and this amount is expected to increase by 50% by 2025 [7]. With a slow degradation rate, CF release absorbed toxins into the soil and waterways, contribute to microplastic pollution, and adversely affect plant growth [7]. Efforts to address this issue include expensive clean-up initiatives and innovative recycling proposals, such as using CF to manufacture bricks [8] and N-doped carbon anodes in lithium-ion batteries [9]. Information on CA degradation is not as widely available, and a gap exists in our understanding of how CA degrades in various environments. A recent study investigated the degradation of CA films in the food packaging industry using anaerobic digestion (AD). However, the maximum final degradation was limited to 74%, due to slow breakdown of CA film [10, 11].

The acetyl moiety of CA, which makes cellulose more hydrophobic and less susceptible to microbial attack is a key factor in its degradation. Notably, previous studies did not explore potential deacetylation procedures that could enhance degradation rates. Therefore, this study focused on the utilization of CA from CF, by aiming to improve enzyme-substrate accessibility through a simpler and more economically efficient pretreatment procedure. Previous studies have emphasized the efficacy of NaOH and KOH in deacetylating CA, particularly in the fabrication of carbon membranes from CA hollow fibers [12]. The investigation with a natural acetylated polymer like β-chitin showed that an increase in NaOH concentration, along with time and temperature, played a significant role in deacetylation efficiency [13]. Another study conducted by Sun et al., achieved 88.3% chitin deacetylation under the optimal experimental condition with NaOH and a high reaction temperature of 100 °C [14].

Therefore, the present study analyzed three thermal alkaline (TA) pretreatments conditions, each varying in time and temperature, to improve CA deacetylation and hydrolysis in an AD system. This helped us to explore microbiome dynamics and propose a breakdown route for CA degradation in an AD system after different pretreatment conditions. The study presents a novel and highly effective pretreatment method and proposes breakdown mechanism for CA degradation under various experimental conditions (control and pretreated) and substrate-specific microbiome development. To the best of our knowledge, this is the first study to achieve the maximum biodegradability of CA (91%) using a feasible deacetylation pretreatment (TA 30 °C). It also provides valuable insight into the microbial community involved in the anaerobic degradation process.

## Materials and methods

### Substrate and inoculum

All experimental samples received inoculation with a culture enriched in a lab-scale thermophilic (60 ℃) seed tank reactor (5 L), initially fed with untreated cigarettes at a concentration of 50 g/L volatile solids (VS). The seed reactor was initially inoculated with municipal sludge (1 L) sourced from the Jungrang municipal treatment facility in Seoul, Korea, characterized by a pH of 7.4 ± 0.1, volatile fatty acids (VFA) at 110.3 ± 28 mg/L, total solids (TS) at 22.6 ± 0.02%, and volatile solids (VS) at 70.7 ± 1.04%. The thermophilic seed tank reactor was consistently maintained within a pH range of 7.5 ∼ 8.0, and the working volume was periodically maintained with a modified basal medium (approximately 1.0 ∼ 1.5 L) containing 50 g/L substrate. This replacement was followed by a complete digestion of the previous substrate loading. With reference to the earlier reports [15], the modified basal media were prepared from the following stock solutions (g of chemicals/L of distilled water): (A) NH_4_Cl, 100; NaC1, 10; MgC1_2_.6H_2_O, 10; CaC1_2_.2H_2_O, 5; (B) K_2_HPO_4_.3H_2_O, 200; (C) trace metal and selenite solution: FeC1_2_.4H_2_O, 2; H_3_BO_3_, 0.05; ZnC1_2_, 0.05; CuCl_2_.2H_2_O, 0.038; MnCl_2_.4H_2_O, 0.05; (NH_4_)_6_Mo_7_O_24_.4H_2_O, 0.05; A1C1_3_, 0.05; CoCl.6H_2_O, 0.05; NiC1_2_.6H_2_O, 0.092; ethylenediaminetetraacetate, 0.5; concentrated HC1, 1 mL; Na_2_SeO_3_.5H_2_O, 0.1; (D) Vitamin solution (mg/L): Biotin, 2; folic acid, 2; pyridoxine acid, 10; riboflavin, 5; thiamine hydrochloride, 5; cyanocobalamin, 0.1; nicotinic acid, 5; P-aminobenzoic acid, 5; lipoic acid, 5; DL-pantothenic acid. The stock solutions were added to 886 mL of distilled water in the following proportions: A (10 mL), B (2 mL), C (1 mL), and D (1 mL). After purging, the additional buffering solution, NaHCO_3_ (2.6 g/L), and reducing agents such as l-cysteine HCl (0.5 g/L) and 0.025% Na_2_S.9H_2_O were added. The CA used in this study was manually separated from popular cigarette brands (ESSE Prime KT and G).

### Pretreatment and characterization of CA

The manually separated CA filters were blended and soaked in a 2% NaOH solution and subjected to three different TA pretreatments TA 30 °C, TA 90 °C, and TA 121 °C incubated at different time periods 24 h, 1 h, and 20 min, respectively. Each pretreated sample was washed twice with distilled water and dried at room temperature to remove the alkali. The chemical alterations resulting from different TA pretreatments were estimated using Fourier transform infrared spectroscopy (FT-IR). Both pretreated and control CA samples were analyzed using a FT-IR Cary 630, Agilent, USA, under specific operational parameters, including 32 scans, 4 resolution, and a wavelength range of 500 ∼ 4,000 cm^-1^.

### Evaluation of anaerobic biodegradability of CA

#### Biomethane potential (BMP)

The biomethane potential (BMP) was experimentally estimated to assess the biodegradability of the control and pretreated CA following a previously reported method [16]. The control experiment, consisting of untreated CA substrate, was included to ensure a clear comparison between the control and pretreated samples. The pretreatment experiments involved three different TA treatments: TA at 30 °C for 24 h, TA at 90 °C for 1 h, and TA at 121 °C for 20 min. The thermophilic BMP test was conducted at 60 °C with a pH range of 7.0–7.5. Each experimental condition was duplicated. The BMP test was carried out in 500 mL-capacity anaerobic bottles with a working volume of 200 mL. To ensure anaerobic conditions, each BMP bottle containing 160 mL of the above mentioned modified basal media was purged with argon gas for approximately 20 min. Subsequently, the anaerobic inoculum (40 mL) from the seed reactor and the respective substrates were introduced into each BMP bottle, which was then perfectly sealed with a silicone stopper and a cap. The additional buffering solution, NaHCO_3_ (2.6 g/L), and reducing agents such as l-cysteine HCl (0.5 g/L) and 0.025% Na_2_S.9H_2_O were added, and the bottles were incubated at 60 °C and 150 rpm for 20 days. Biogas production, VFAs, and VS were measured as described previously [17]. The biogas analysis was conducted using Gas Chromatography with a Thermal Conductivity Detector (GC-TCD). Additionally, liquid samples for VFAs estimation were collected every three days, with each sample containing 1 mL. VFAs in the experimental samples were analyzed using a gas chromatograph (GC; Agilent 6890 N, USA) equipped with a flame ionization detector (FID), followed a previously established procedure [18]. The VFA yield (g/g of VS) was calculated as the total VFAs produced per gram of VS during the BMP period.

#### Theoretical biomethane potential (tBMP)

To determine the theoretical biomethane potential (tBMP) of the CA, its elemental composition was analyzed using a Flash Smart Elemental Analyzer (Thermo Scientific Inc, USA). This analyzer operates through dynamic flash combustion, followed by the separation and detection of resulting gases to measure carbon (C), hydrogen (H), nitrogen (N), sulfur (S), and oxygen (O) percentages. Approximately 5 mg of dried CA sample was placed in a tin or silver container and introduced into the combustion reactor via an autosampler. For CHNS analysis, the sample underwent combustion at 950 °C with copper oxide and electrolytic copper in the presence of helium and excess oxygen. This process elevated the temperature to around 1800 °C, converting all elements into their respective gaseous oxides: carbon to carbon dioxide (CO₂), hydrogen to water (H₂O), nitrogen to nitrogen gas (N₂) or nitrogen oxides (NxOY), and sulfur to sulfur dioxide (SO₂) and sulfur trioxide (SO₃). The gases then passed through electrolytic copper to trap excess oxygen, yielding N₂, CO₂, H₂O, and SO₂. After purification with a helium carrier gas through traps and scrubbers to remove impurities, the gases underwent chromatographic separation based on different retention times in a GC-column. TCD measured changes in thermal conductivity as gases passed through, generating signals proportional to the gas amounts. These signals were processed and converted into the percentage composition of C, H, N, and S using calibration curves derived from standards.

For oxygen analysis, the dried CA sample was placed in a silver container and combusted at 1060 °C in a reactor containing nickel-plated carbon and quartz turnings. The resulting gases, including carbon monoxide (CO) and N₂, were directed through quartz turnings and an absorbent filter before separation in GC columns based on retention times. The TCD detected these gases, generating signals used to calculate the O % in the original sample.

This analytical method provides crucial data on the elemental composition of the CA sample, which is essential for determining its tBMP. tBMP was then calculated based on the elemental composition of the experimental substrates, as shown in Table 1, using Buswell and Müller’s equations in Eq. (1) [17].

**Table 1:** Examined CA substrate in BMP for methane production, and observed biodegradability with various pretreatments

Condition	Elemental composition			VS^a^	tBMP^b^	BMP^c^	VFAs^d^ Yield (g/g of VS^a^)	Observed Biodegradability (%)
	Carbon (%)	Hydrogen (%)	Oxygen (%)	(%)	(mL/g of VS^a^)	(mL/g of VS^a^)		
Control	48.3	5.8	41.5	99.7	487.0	306.0 ± 10	0.2	63
TA 30°C	46.0	6.0	45.3	99.2	762.6	695.0 ± 4	0.2	91
TA 90°C	47.0	5.6	45.5	99.0	777.3	639.6 ± 7	0.3	82
TA 121°C	43.1	5.7	45.6	98.4	768.6	437.7 ± 8	0.8	57

^a^Volatile solids, ^b^theoretical biomethane potential, ^c^experimental biomethane potential, and ^d^volatile fatty acids. Observed Biodegradability = (BMP/tBMP) *100

1 $$\begin{aligned}&{\varvec{C}}_{\varvec{c}}{\varvec{H}}_{\varvec{h}}{\varvec{O}}_{\varvec{o}}{\varvec{N}}_{\varvec{n}}{\varvec{S}}_{\varvec{s}}+\left(\varvec{c}-\frac{\varvec{h}}{4}-\frac{\varvec{o}}{2}+\frac{3\varvec{n}}{4}+\frac{\varvec{s}}{2}\right){\varvec{H}}_{2}\varvec{O}\\ &\to\:\left(\frac{\varvec{c}}{2}+\frac{\varvec{h}}{8}-\frac{\varvec{o}}{4}-\frac{3\varvec{n}}{8}-\frac{\varvec{s}}{4}\right)\varvec{C}{\varvec{H}}_{4}\\&+\left(\frac{\varvec{c}}{2}-\frac{\varvec{h}}{8}+\frac{\varvec{o}}{4}+\frac{3\varvec{n}}{8}+\frac{\varvec{s}}{4}\right)\varvec{C}{\varvec{O}}_{2}+\varvec{n}\varvec{N}{\varvec{H}}_{3}+\varvec{s}{\varvec{H}}_{2}\end{aligned}$$

### Observed biodegradability

The observed biodegradability of CA was calculated as shown in equation (Eq. (2). [19, 20]

2 $$\:\varvec{O}\varvec{b}\varvec{s}\varvec{e}\varvec{r}\varvec{v}\varvec{e}\varvec{d}\:\varvec{B}\varvec{i}\varvec{o}\varvec{d}\varvec{e}\varvec{g}\varvec{r}\varvec{a}\varvec{d}\varvec{a}\varvec{b}\varvec{i}\varvec{l}\varvec{i}\varvec{t}\varvec{y}\:\left(\mathbf{\%}\right)=\left(\frac{\varvec{B}\varvec{M}\varvec{P}}{\varvec{t}\varvec{B}\varvec{M}\varvec{P}}\right)\times\:100$$

Here, BMP represents the actual methane production observed during the experimental period, while tBMP denotes the maximum methane production expected from complete biodegradation of CA. By dividing BMP by tBMP and multiplying by 100, the equation expresses the fraction of CA that has been biodegraded, providing a measure of the material’s degradability under the specific experimental conditions.

### Microbiome community analysis

All experimental CA samples were subjected to microbiome community analysis targeting both bacteria and archaea. DNA was extracted using the Fast DNA^™^ Spin Kit for Soil (MP Biomedicals, LLC, Solon, OH, USA) following the manufacturer’s protocol. The concentration of the extracted double-stranded DNA was determined using an Infinite M200 PRO microplate reader (Tecan Austria GmbH, Grödig, Austria). The extracted DNA was submitted to CJ Biosciences, Inc. (Seoul, South Korea) for sequencing, using an Illumina MiSeq platform (San Diego, CA, USA).

For the Illumina platform preparation, both forward and reverse fusion primers were used. Specifically, the V3–V4 region of the bacterial 16S rRNA gene in each sample was amplified using primers 341 F and 805 R. Additionally, the methanogen-specific primers Arch519F (CAGCCGCCGGTAA) and Arch934R (GTGCTCCCCCGCCAATTC) were used to detect the methanogen community. Dr. MAX DNA polymerase (Doctor Protein Inc., Korea), 10 pmol of each primer, and 1 µL of template were used for amplifications in a 25 µL reaction volume. The thermal cycles included an initial step at 95 °C for 3 min, followed by 25 cycles at 95 °C, 55 °C, and 72 °C for 30 s each, and a final cycle at 72 °C for 5 min. Following the preparation of raw sequence reads, compositions and amounts of bacteria in shared sets of several samples were examined using the CL community (Version 3.46, CJ Bioscience., Seoul, Korea). Using the EZbiocloud database, each sequence was processed and taxonomically allocated. Bacterial and methanogen community distributions at the phylum and genus levels were represented by R (ggplot) and Circos.

## Results

### Characterization of CA under different pretreatment conditions

**Fig. 1:**
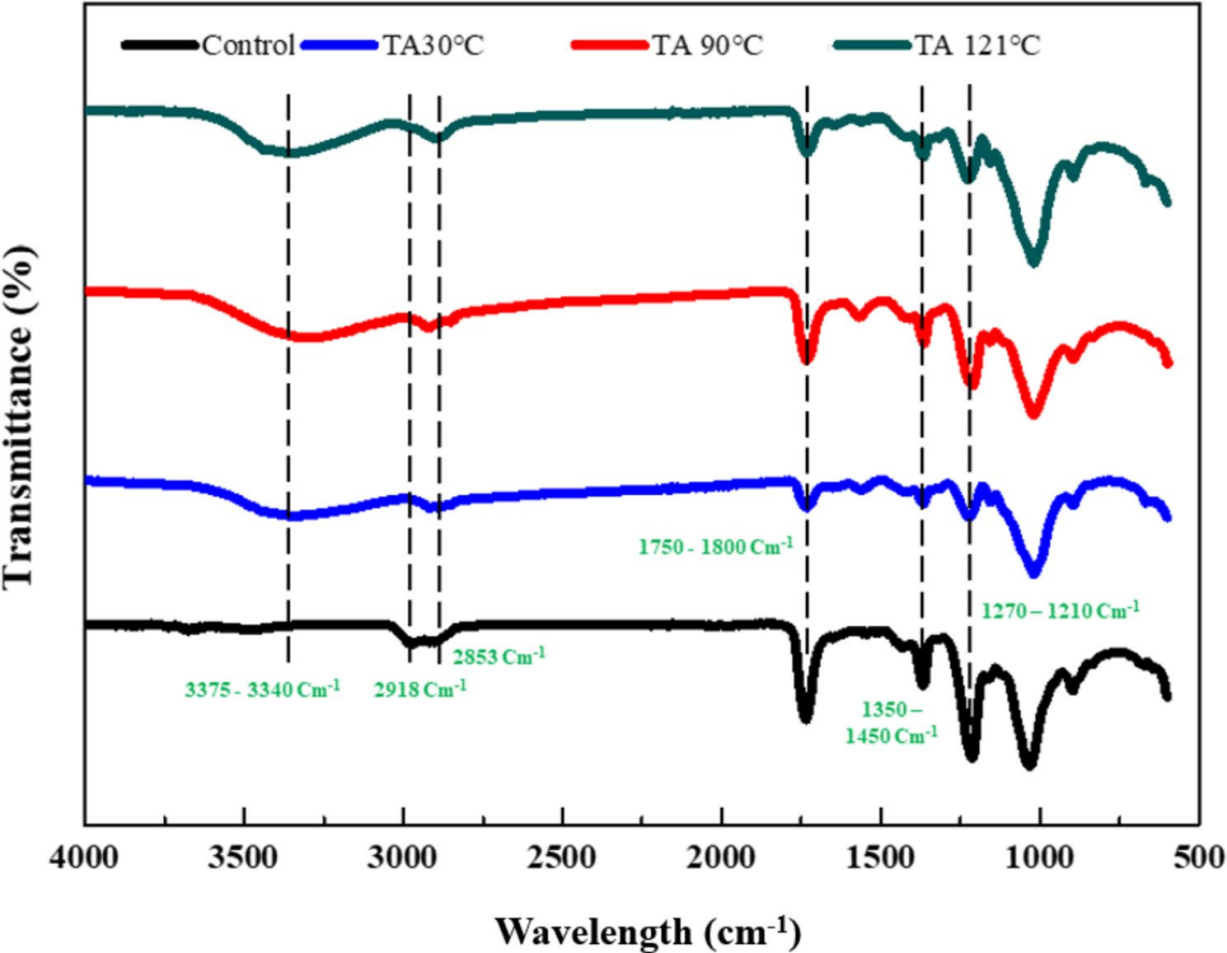
Effects of thermal alkaline (TA) pretreatments on cellulose acetate (CA): Major structural changes induced in the CA molecule by each pretreatment are indicated by dotted lines

Fourier-transform infrared (FT-IR) analysis revealed the structural changes in CA caused by the different TA pretreatments. As shown in Fig. 1, the untreated control CA exhibited functional groups at wavelengths of 3,375 ∼ 3,340 cm^− 1^ [21], 2,918 cm^− 1^ [22], 2,853 cm^− 1^ [22], 1,750 ∼ 1,800 cm^− 1^ [23], 1,350 ∼ 1,450 cm^− 1^ [24, 25] and 1,270 ∼ 1,210 cm^− 1^ [22]. However, all the TA-pretreated samples exhibited a clear reduction in the C = O band at 1,750 ∼ 1,800 cm^− 1^ [23], implying significant changes in the chemical structure. Furthermore, all the pretreatment samples showed some changes in the C − O stretch (1,270 ∼ 1,210 cm^− 1^) [22], C-H stretching vibrations (2,853 cm^− 1^ and 2,918 cm^− 1^) [22], and the appearance of a broad peak around 3,375 ~ 3,340 cm^− 1^ wavelengths [21].

### Biodegradability of CA

Table 1 shows the biodegradability of CA after various TA pretreatments. The BMP of the pretreated CA samples was assessed to evaluate their capacities for both energy production and biodegradation. The control sample exhibited a BMP of 306.0 ± 10 mL/g VS. Notably, TA at 30 °C and 90 °C conditions showed significantly higher methane production, accumulating 695.0 ± 4 mL/g VS and 639.6 ± 7 mL/g VS, respectively. The VFA yield was 0.2 and 0.3 g/g of VS for TA 30 °C and TA 90 °C conditions, respectively. In contrast, the TA 121 °C condition resulted in lower methane production (437.7 ± 8 mL/g VS) but higher VFA yield (0.8 g/g of VS). The biodegradability of CA after pretreatment was determined by dividing the experimental BMP value by the tBMP value. The tBMP value was calculated based on the elemental composition (C, H, and O%) using the Buswell and Müller equation. The biodegradability of the control sample was 63%, whereas the biodegradability of the TA pretreated samples was higher. TA 30 °C conditions showed the maximum biodegradability 91%, followed by TA 90 °C with 82% and TA 121 °C with 57% biodegradability.

### Microbiome community distribution during CA degradation

**Fig. 2:**
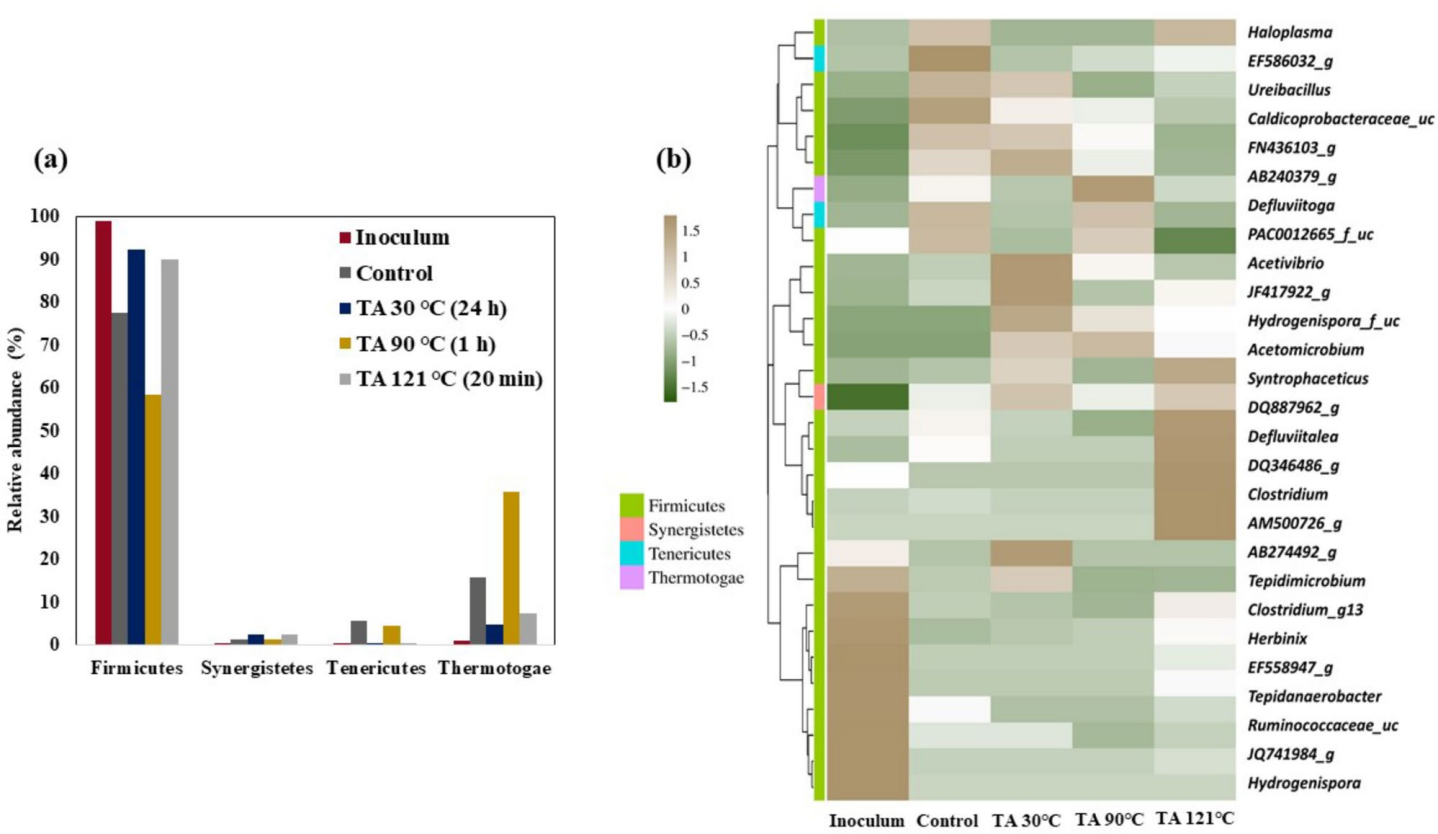
Bacterial community analysis during cellulose acetate (CA) degradation: (**a**) the relative abundance of bacterial phyla in each experimental condition. (**b**) Cluster mapping of bacterial taxonomy at the genus level with > 1% relative abundance. Following the Euclidean distance matrix, the degree of correlation between the respective genus and experimental condition is represented by the color of the boxes. (green boxes represent the negative correlation (-1.5), white colored boxes represent a neutral correlation (0.0), and brown boxes show a high correlation (1.5) between the respective genus and experimental condition)

The bacterial and methanogenic communities were taxonomically classified and studied at both the phylum and genus levels for all experimental samples collected during the time of peak CH_4_ production. Figure 2a illustrates the classification of bacterial phyla across all experimental conditions, including the inoculum, untreated control, and TA-pretreated conditions. Firmicutes emerged as the dominant phylum in all conditions, with a notable abundance of Thermotogae in both the control and the TA 90 ℃ condition. Synergistetes and Tenericutes were less abundant across all conditions, with Tenericutes being exclusively found in the control and the TA 90 ℃ condition. Figure 2b shows cluster correlation mapping, which employs the Euclidean distance metric to represent the genus-level classification of bacterial taxonomy. In the first cluster (from below), a significant correlation was observed among genera belonging to the phylum Firmicutes. Specifically, genera such as *Hydrogenospira*,* JQ741984_g*,* Ruminococcaceae_uc*,* Tepidanaerobacter*,* EF558947_g*,* Herbinix*,* Clostridium_g13*,* Tepidimicrobium*, and *AB274492_g (Caldicoprobacteraceae*) were strongly correlated with the inoculum samples. Within the second cluster, the TA 121 °C pretreatment shows a strong correlation with certain genera, namely, *Clostridium*,* DQ346486_g*,* Defluvittalea*,* DQ887962_g*,* Syntrophaceticus*,* Acetomicrobium*, and *Hydrogenispora_f_uc.* Interestingly, some high to moderate correlated genera present in this cluster, including *Acetomicrobium*,* Hydrogenispora_f_uc*,* JF417941_g*,* Thermoacetogenium*,* Coprothermobacter*, and *JF417922_g* were consistently found under all the TA pretreatment conditions. Similarly, in the third cluster, a strong correlation was observed with the control samples. However, certain genera, such as *Acetovibiro*,* PAC001265_f_uc*,* Defluvittoga*,* AB240379_g*,* FN436103_g*, *Caldicoprobacteraceae_uc*,* Urebacillus*, and *EF586032_g*, displayed moderate to high correlation with TA 30 °C and TA 90 °C conditions. *Haloplasma* shares a strong correlation with control and TA 121 °C. A notable observation is that the microbial distribution of the TA 30 °C condition overlaps with all three clusters, indicating a high level of diversity within this specific pretreatment.

**Fig. 3:**
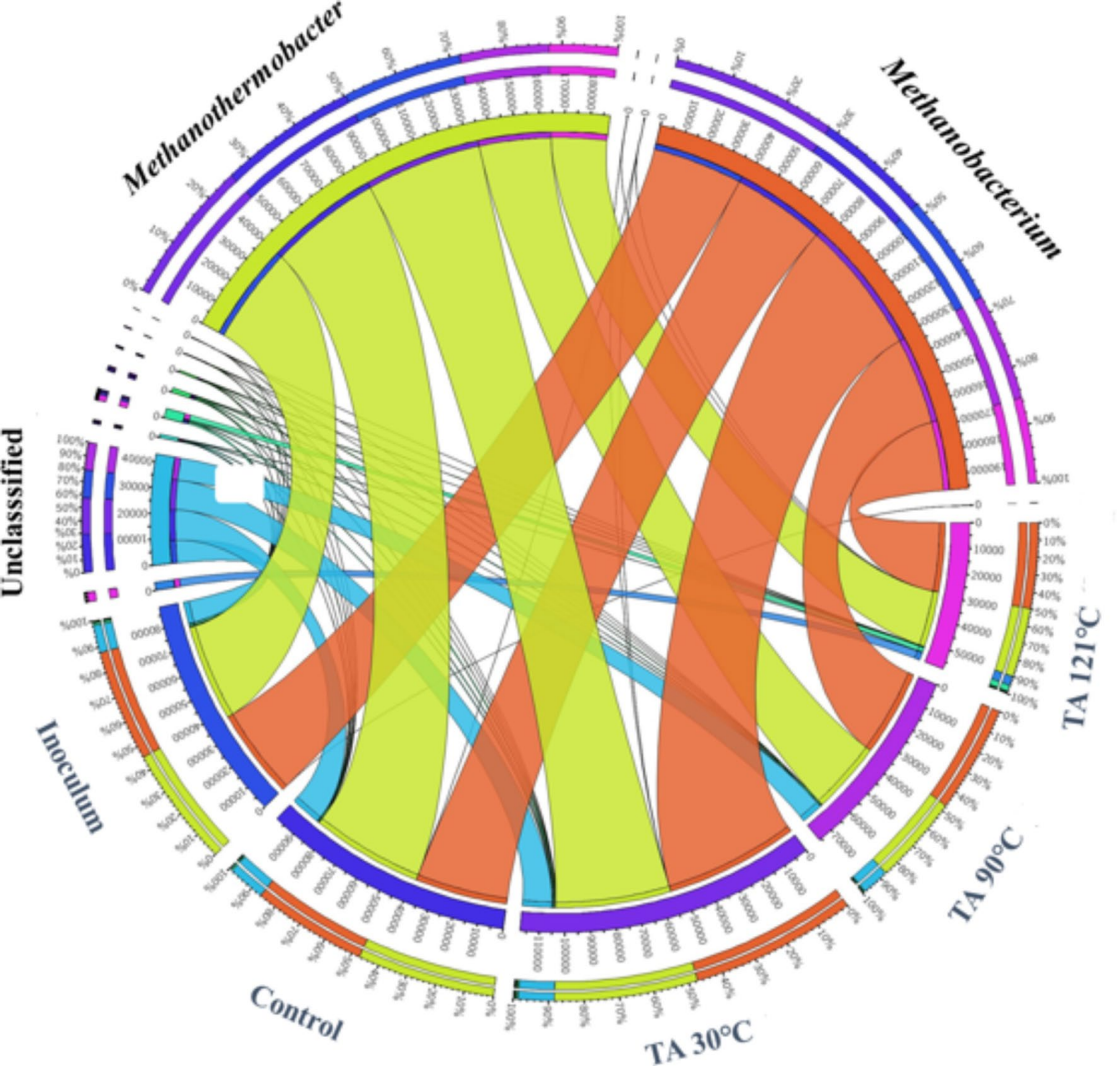
Methanogen Community analysis during cellulose acetate (CA) degradation: Circos representation of archaeal taxonomy at the genus level. The relative abundance of each methanogen genus under each pretreatment condition (Blue labels) is described

The archaeal community, under all experimental conditions, comprised the Euryarchaeota phylum (data not shown). The genus-level classification of the archaeal community is represented in a Circos figure (Fig. 3). This figure clearly illustrates the dominance of two hydrogenotrophic methanogen genera, *Methanobacterium* and *Methanothermobacter* across all experimental conditions and inoculum.

## Discussion

### TA pretreatment induced chemical changes for enhanced biodegradability of CA

Bioplastic biodegradability depends on various physical and chemical properties, such as hydrophilicity, crystallinity, and elemental composition [26]. CA is a synthetic polymer derived from cellulose; therefore, it is vital to increase its hydrophilicity and substrate accessibility by deacetylation.

Pretreatment of CA with NaOH for AD is not a common practice. However, NaOH is widely used with other AD substrates like corn stover to significantly increase biogas yield by enhancing substrate breakdown and removing lignin and other inhibitors. Previous study showed that pretreatment with high concentrations of NaOH (7.5%) in corn stover can lead to rapid VFA production, which inhibits subsequent stages of AD [27]. Conversely, another study with corn stover showed that pretreatment with low NaOH concentration (2%) is effective for AD process, improving biogas production [28]. Based on these insights, we decided to use 2% NaOH for our CA pretreatment. Previous studies also support that 0.2 M NaOH solution could effectively deacetylate CA hollow fiber membranes, resulting in chemical structural changes. Our observations confirmed these findings [12].

To elucidate the specific chemical changes in CA resulting from pretreatment, we analyzed FTIR. As seen in Fig. 1, the C = O stretching vibration peak associated with the ester groups in CA (1,750 ∼ 1,800 cm^− 1^) is weaker in the TA pretreated samples than in the control CA sample [23]. This suggests that the TA pretreatment removed some ester groups from the CA molecule. Additionally, the relative absorption intensities of the C − O stretching, in the range of 1,270 ∼ 1,210 cm^− 1^, corresponding to the acetyl groups, were decreased in all pretreatment samples [22]. Moreover, the C-H bending vibration peak in the methyl and methylene groups (1,350 ∼ 1,450 cm^− 1^) was weaker in the TA-pretreated samples than in the control CA sample [24, 25]. This indicates that TA pretreatment breaks down the CA molecules into smaller chains. Eventually, the broad peaks at approximately 3,375 ∼ 3,340 cm^− 1^ wavelength indicating the stretching vibration of free OH groups in cellulose after deacetylation pretreatment in CA [21]. The previous studies state that the deacetylation of CA with NaOH involves breaking the ester bonds between the acetyl groups and the cellulose backbone, converting the material into cellulose. When CA is treated with NaOH, the hydroxide ions (OH−) from the NaOH solution attack the carbonyl carbon of the ester bonds in the hydrophobic acetyl groups attached to the cellulose chain. This nucleophilic attack forms a tetrahedral intermediate, which then breaks down, resulting in the cleavage of the ester bond. The acetyl group is released as an acetate ion (CH_3_COO−), and a hydrophilic hydroxyl group (-OH) is added to the cellulose backbone, effectively regenerating the cellulose from CA [29]. The overall reaction converts CA into cellulose and sodium acetate (CH_3_COONa), with the NaOH facilitating the removal of the hydrophobic acetyl groups and restoring the hydrophilic hydroxyl groups on the cellulose. This transformation increases the hydrophilicity of the cellulose, making it more accessible for enzymatic hydrolysis and microbial degradation in anaerobic digestion processes. In that regard, the observed chemical structural changes in this study indicate efficient deacetylation of CA substrate after TA pretreatment [30].

### Impact of TA pretreatment on the biodegradation rate and elemental composition of CA

In this study, experiments were conducted to assess the effects of pretreatment on the BMP and biodegradability of CA. The biodegradability of pretreated CA was compared with that of the control samples to identify the optimal pretreatment conditions and enhance CA conversion into biogas. Compared with the control, pretreatment with TA resulted in a significant increase in methane production rate (Table 1). The pretreatments TA 30 °C and TA 90 °C yielded high methane production rate (695.0 ± 4 mL/g VS and 639.6 ± 7 mL/g VS, respectively), compared to 306.0 ± 10 mL/g VS for the control. From Table 1, the VFA yields were also appropriately enhanced for the pretreated samples 0.3 g/g of VS for TA 90 °C compared to the control with 0.2 g/g VS. This suggests that pretreatment makes CA more amenable to biodegradation, enhances accessibility to microorganisms, and improves the efficiency of VFA conversion to methane. In contrast, pretreatment with TA 121 °C resulted in a low methane production rate (437.7 ± 8 mL/g VS) and a high VFA accumulation rate (0.8 g/g VS), indicating that this pretreatment resulted in conditions that failed to convert the available VFAs to biogas products. The large variation in oxygen percentage resulting from the TA pretreatments greatly influenced the tBMP value. These results aligned with the high deacetylation effect observed in the various TA pretreatments. Compared to the control, the enhanced biodegradability of CA with different TA pretreatment conditions improved the overall biodegradation potential. Moreover, after TA 30 °C pretreatment, the rate of CA biodegradability increased to 91%, the highest biodegradability of CA from any waste source previously reported.

### Microbial community dynamics and functional roles in CA degradation with different pretreatments

Firmicutes dominated under all conditions, and Thermotogae was the second most abundant phylum in all experimental samples, excluding the inoculum (Fig. 2a). This demonstrates that Thermotogae comprises a selective CA-degrading microbiome whose growth is aided by highly amorphous (control) and TA-aided highly amorphous deacetylated CA substrates. In both the TA 30 ℃ and TA 90 ℃ conditions, the Firmicutes and Thermotogae played a synergetic role by enriching the AD system with hydrolyzing and acetogenic bacteria. Synergistetes and Tenericute*s* are the two other phyla that play minor roles in CA degradation.

In the first cluster (Fig. 2b), the majority of highly correlated genera, such as *Hydrogenispora*,* JQ741984_g (Caldicoprobacteraceae_f)*,* Ruminococcaceae_uc*,* Tepidanaerobacter*,* EF558947_g*,* Herbinix*,* Clostridium_g13*,* Tepidimicrobium*, and *AB274492_g (Caldicoprobacteraceae_f)*, were primarily associated with inoculum conditions. Remarkably, the inoculum also exhibited a prevalence of hydrolytic bacteria, including *Clostridium_g13* and *Herbinix*, as well as acetate-oxidizing bacteria such as *Tepidanaerobacter* [31, 32]. Additionally, *EF558947_g* (*Xylanivirgaceae_f*) possesses the capability to utilize microcrystalline cellulose and d-cellobiose as energy sources [33]. Cellulose breakdown by bacteria from the family *Xylanivirgaceae*, involves a series of enzymatic reactions that enable these organisms to degrade cellulose efficiently. Initially, these bacteria adhere to cellulose fibers, facilitating enzymatic action. They produce a suite of cellulolytic enzymes including endoglucanases, exoglucanases (cellobiohydrolases), and β-glucosidases. Endoglucanases cleave internal bonds within the cellulose chain, generating shorter oligosaccharides. Exoglucanases act on the chain ends, releasing cellobiose units, which are further hydrolyzed by β-glucosidases into glucose monomers. Cellobiose phosphorylase also plays a critical role by converting cellobiose into glucose-1-phosphate and glucose, which can be metabolized for energy production and growth. This enzymatic synergy ensures the complete breakdown of cellulose into fermentable sugars like glucose and cellodextrins. These sugars are then utilized by the *Xylanivirgaceae* to produce VFAs byproducts like acetic acid, propionic acid, and butyric acid via glycolysis and various fermentative pathways [34].

Unlike other genera in the cluster, *AB274492_g (Caldicoprobacteraceae)*, a bacterium with the capacity to break glucose into VFA, ethanol and contribute CO_2_ and H_2_ production [35–37], still maintained a moderate level of correlation, particularly in the TA 30 °C condition.

The genera present in the first clade of the second cluster (Fig. 2b) exhibit a strong correlation with the TA 121 °C condition. This association is significant because it signifies the effective hydrolysis of cellulose and cellobiose, a process facilitated by bacteria such as *Defluviitalea*, and *Clostridium* [38–40]. Furthermore, the TA 121 °C condition was characterized by the abundance of other microorganisms with specific metabolic functions. These include VFA-oxidizing bacteria and alcohol-consuming bacteria, such as *DQ346486_g (Limnochordaceae_f)*,* Syntrophaceticus* [41], and *DQ887962_g* [42]. The presence of these microorganisms is consistent with the high concentration of VFA (a final VFA concentration of 3.8 g/L of VS). Additionally, two other genera, *namely*,* Hydrogenispora_f_uc*, and *Acetomicrobium*, are moderately correlated in the control and TA 30 °C conditions. *Acetomicrobium* is known for its ability to ferment glucose and cellobiose, contributing to their metabolism [43]. However, *Hydrogenispora_f_uc* appeared to have some adverse effects, with *Hydrogenispora_f_uc* involved in hydrogen fermentation. The four other genera present in the cluster, namely, *JF417941_g (Limnochordaceae_f)*,* Thermoacetogenium*, *Coprothermobacter*, and *JF417922_g (Thermoanaerobacterales_O)* hold a strong to moderate association with both TA 30 °C and TA 90 °C conditions. Although *JF417922_g* thrives on glucose or cellobiose substrates, the other three acetogens play effective roles in acetate oxidation, hydrogen fermentation, and ethanol consumption.

The first clade of final cluster (Fig. 2b) showed a strong correlation with both the control and TA 90 °C conditions. Within this cluster, the presence of *Acetivibrio* is noteworthy because it can ferment cellulose and glucose and produce short-chain fatty acids (SCFAs), including acetate. In contrast, *Defluviitoga* appears to feed on the cellobiose and arabinose released during the cellulose breakdown. Additionally, it serves as a sulfate-reducing bacterium, participating in sulfate-reduction processes [44]. In contrast, *PAC001265_f_uc (Mollicutes_c)* cannot break down complex sugars such as cellulose, limiting their activity in glucose fermentation [45].

The remaining genera in the final cluster are strongly correlated with both control and TA 30 °C conditions, wherein, the, *AB240379_g (Oscillospiraceae_f)*,* FN436103_g (Turicibacteraceae_f)*,* and Caldicoprobacteraceae_uc* can effectively degrade cellulose and other complex carbohydrates, and produce metabolic byproducts, such as SCFAs. *EF586032_g (Mollicutes_c)* shares similar activity as *PAC001265_f_uc* and *Haloplasma* adapting to extreme environmental conditions, suggesting potential challenges or limitations associated with their metabolic activities.

As shown in Fig. 3, only hydrogenotrophic methanogens, namely, *Methanothermobacter* and *Methanobacterium* prevailed under all conditions. Earlier studies [46] reported that *Methanobacterium* initially form aggregates and release CH_4_ by absorbing H_2_ and CO_2_ as their only energy sources. *Methanothermobacter*, the second most dominant methanogen in our study, can effectively reduce CO_2_ with H_2_ and acetic acid, and formic acid as potential terminal electron acceptors to produce CH_4_ [47].

### Prediction of a CA breakdown pathway

**Fig. 4:**
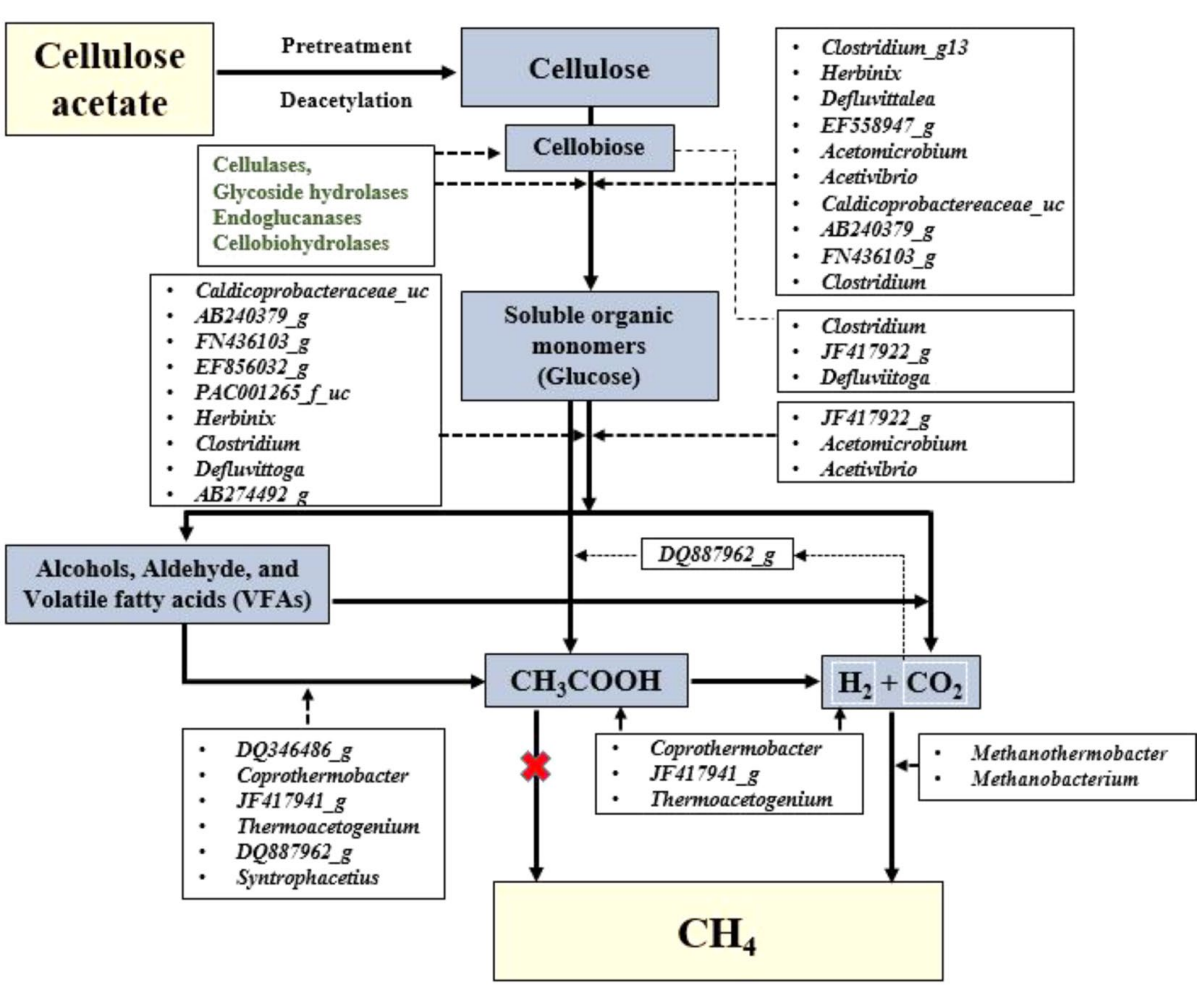
Cellulose acetate (CA) breakdown pathway prediction: Dominant thermophilic hydrolytic, acidogenesis, acetogenesis bacteria, and hydrogenotrophic methanogens involved in the breakdown pathway. The greyish-blue color represents the by-products of each anaerobic digestion (AD) stage; the green color symbolizes the hydrolytic enzymes involved; the white box represents the dominant microbiomes involved in each AD process

Currently, the degradation of bioplastics via four critical AD phases is generally recognized and accepted by many researchers. Therefore, this study mapped the dominant bacterial and archaeal groups to three critical stages of CA degradation, proposing a breakdown pathway for high-temperature CA degradation (Fig. 4). Prior to the BMP process, the CA substrates were deacetylated to cellulose using time- and temperature-varying TA pretreatment strategies. Based on their deacetylation efficiency, the experimental conditions enriched substrate-specific (control and TA-pretreated) microbiomes at different AD phases (i.e., extended or shortened hydrolysis phases), resulting in distinct degradation patterns. This justifies the inclusion of all prominent microbiomes in the CA breakdown pathway, irrespective of their deacetylation efficiency.

The initial stage involved hydrolysis and fermentation. During this stage, cellulose, with a certain amount of non-deacetylated CA substrate and cellobiose, is broken down into soluble simple sugars, such as glucose. In the present study (Fig. 4), *Clostridium_g13*,* Herbinix*,* Defluvittalea*,* EF558947_g*,* Acetomicrobium*,* Acetivibrio*,* Caldicoprobactereaceae_uc*,* AB240379_g*,* FN436103_g*,* and Clostridium* could hydrolyze cellulose, whereas some species of *Clostridium*,* JF417922_g*,* and Defluviitoga* were limited to cellobiose degradation. These hydrolyzing bacteria produce various enzymes such as cellulases, endoglucanases, cellobiohydrolases, and glycoside hydrolases, which convert insoluble organic components into soluble organic sugar monomers. During the fermentation process, bacteria (e.g., *JF417922_g*,* Acetomicrobium*,* Acetivibrio*,* Caldiprobacteraceae_uc*,* AB240379_g*,* FN436103_g*,* EF856032_g*,* and PAC001265_f_u*) ferment glucose to produce acids, aldehydes, alcohols, H_2_, and CO_2_. Interestingly, some hydrolyzing bacteria (such as *Herbinix*,* Clostridium*, and *Defluviitoga*) are also involved in fermentation.

In the next stage, certain acetogens aid in the degradation of SCFAs, alcohols, and aldehydes from hydrolytic fermentation to acetic acid and H_2_. In our study, we found *AB274492_g* could effectively utilize these by-products and produce hydrogen, whereas other acetogens, such as *DQ346486_g*,* Coprothermobacter*,* JF417941_g*, *and Thermoacetogenium* can aid in acetic acid production. *DQ887962_g* and *Syntrophaceticus* are known for their propionate-oxidizing activities. Interestingly, *DQ887962_g* converts carbohydrate monomers to acetic acid in the presence of CO_2_.

Generally, during the final stage of methanogenesis, methane is produced by both acetoclastic and hydrogenotrophic methanogens. However, in our inoculum and experimental condition, we observed only abundance of hydrogenotrophic methanogens. Hydrogenotrophic methanogens, such as *Methanobacterium* and *Methanothermobacter* use H_2_ and CO_2_ to form CH_4_. This finding is of interest because in the TA 121 °C condition, even though there are numerous acetogens (VFAs and acetate-oxidizing bacteria) such as *Thermoacetogenium*,* Coprothermobacter*,* JF417941_g*,* DQ887962_g*,* Syntrophacetius*, and *DQ346486_g* with a limited presence of hydrolytic bacteria (*Clostridium* and *Defluvittoga*) (Fig. 2b). Based on these results, we hypothesize that TA 121 °C pretreatment could have enhanced the cellulose degradation and produced lower molecular weight cellulosic oligomers, which can be rapidly hydrolyzed and produce acetic acid and other VFA’s. The high VFA yield (0.8 g/g of VS) observed in BMP experiments (with TA 121 °C samples) resulted in VFA accumulation. This may lead to the inhibition of methanogen proliferation, leading to overall instability in the AD system. However, the other two TA pretreatment conditions resulted in the proliferation of a microbiome that favored degradation. In the TA 30 °C condition (Fig. 2b), the microbiome community was diverse with hydrolytic (*FN436103_g*,* AB240379_g*,* Caldiprobacteraceae_uc*,* JF417922_g*, and *Acetomicrobium*), acidogenic (*FN436103_g*,* AB240379_g*,* Caldiprobacteraceae_uc*,* Acetomicrobium*, and *AB274492_g)*, acetogenic (*Thermoacetogenium*,* Coprothermobacter*, and *JF417941_g*), and methanogenic (*Methanobacterium* and *Methanothermobacter*) bacteria (Fig. 3). TA 90 °C condition (Fig. 2b) had higher abundance of hydrolytic (*FN436103_g*,* Acetivibrio*,* Defluvittoga*,* JF417922_g*, and *Acetomicrobium*), and sufficient acidogenic (*PAC001265_f_uc*,* Acetivibrio*,* Defluvittoga*,* JF417922_g*, and *Acetomicrobium)* and acetogenic (*Thermoacetogenium* and *JF417941_g*), and methanogenic (*Methanobacterium* and *Methanothermobacter*) bacteria (Fig. 3).

## Conclusion

This study addresses the waste management challenges associated with bioplastics and emphasizes the need for a cost-effective waste management approach. Recognizing the issue of microplastic accumulation in bioplastic degradation research, the study focuses on enhancing substrate degradability using a simple and economically viable pretreatment (TA 30 °C), achieving a maximum CA degradation of 91% with optimal removal of acetyl groups (C = O band at 1,750 ∼ 1,800 cm^− 1^, C-H bending vibration at 1,350 ∼ 1,450 cm^− 1^, and C–O stretch at 1,270 ∼ 1,210 cm^− 1^) of CA. This study focused on the degradability of CA sourced from CF, one of the most common and abundant types of plastic waste worldwide. The active involvement of a diverse microbiome community, including *AB240379_g*,* Acetomicrobium*,* FN436103_g*,* JF417922_g*,* AB274492_g*,* Coprothermobacter*,* Methanobacterium*,* Methanothermobacter*, etc., is highlighted, and their potential roles in CA degradation are elucidated.

## Data Availability

No datasets were generated or analysed during the current study.

## Abbreviations

CA: Cellulose Acetate
CF: Cigarette Filters
VS: Volatile Solids
VFA: Volatile Fatty acids
TS: Total Solids
TA: Thermal Alkaline
FTIR: Fourier Transform Infrared Spectroscopy
BMP: Biomethane Potential
tBMP: Theoretical Biomethane Potential
SCFA: Short Chain Fatty Acid
